# Effects of cardiac surgical support on long-term outcomes of emergent or complex percutaneous coronary intervention cases: a sub-analysis of the SHINANO 5-year registry

**DOI:** 10.1007/s00380-021-02015-6

**Published:** 2022-01-07

**Authors:** Chie Nakamura, Soichiro Ebisawa, Takashi Miura, Hidetomo Nomi, Yusuke Kanzaki, Hisanori Yui, Shusaku Maruyama, Ayumu Nagae, Yasushi Ueki, Takahiro Sakai, Tamon Kato, Tatsuya Saigusa, Ayako Okada, Hirohiko Motoki, Koichiro Kuwahara

**Affiliations:** 1 Department of Cardiovascular Medicine, Shinshu University School of Medicine, 3-1-1 Asahi, Matsumoto-shi, Nagano, 390-8621 Japan; 2 Department of Cardiology, Nagano Municipal Hospital, Nagano, Japan

**Keywords:** Coronary artery disease, Cardiac surgery, Prognosis, Percutaneous coronary intervention

## Abstract

Significant improvements in percutaneous coronary intervention (PCI) technology have enabled cardiovascular procedures to be performed without onsite cardiac surgery facilities. However, little is known about the association between onsite cardiac surgical support and long-term outcomes of PCI, particularly among emergent and complex cases. We investigated whether the presence or absence of cardiovascular surgery affects the long-term prognosis after PCI, emergent and complex elective cases. The SHINANO 5-year registry, a prospective, observational, and multicenter cohort study registry in Nagano, Japan, consecutively included 1665 patients who underwent PCI between August 2012 and July 2013. The procedures were performed at 11 hospitals with onsite cardiac surgery facilities [onsite surgery (+) group; *n* = 1257] and 8 hospitals without onsite cardiac surgery facilities [onsite surgery (−) group; *n* = 408]. The primary endpoint was all-cause mortality and the secondary endpoint was major adverse cardiac and cerebrovascular events [MACCE: all-cause death, Q-wave myocardial infarction, non-fatal stroke, and target lesion revascularization]. The onsite surgery group (+) had a lower rate of emergent PCI and ST-segment elevation myocardial infarction (40.8% vs. 51.7%, *p* < 0.01 and 24.9% vs. 39.2%, *p* < 0.01, respectively), and a higher prevalence of hemodialysis and history of peripheral artery disease (7.6% vs. 2.45%, *p* < 0.01 and 12.1% vs. 6.9%, *p* < 0.01, respectively). However, the Kaplan–Meier analysis showed no difference in the 5-year mortality rate (16.4% vs. 15.2%, *p* = 0.421) and MACCE incidence (31.6% vs. 28.9%, *p* = 0.354) between the groups. Also, there were no differences in the mortality rate and incidence of MACCE among emergent cases of ST-segment elevation myocardial infarction and complex elective cases who underwent PCI. Long-term outcomes of PCI appear to be comparable between institutions with and without onsite cardiac surgical facilities.

## Introduction

Percutaneous coronary intervention (PCI) centers without onsite cardiac surgical support are currently available. With significant improvements in PCI technology and devices, the initial procedural outcome and incidence of complications have improved [1–7]. In addition, since PCI has become an effective treatment modality for coronary artery disease (CAD), it is possible to provide rapid treatment for patients living in areas away from hospitals that offer cardiovascular surgery. In 2005, it was shown that 16% of all PCI centers in the United States operated without onsite backup cardiac surgery facilities, and this rate may have increased in recent years [8].

Large meta-analyses, prospective registry studies, single-center studies, and retrospective studies have reported no significant differences in mortality rate between the two types of institutions (regarding the availability or unavailability of cardiovascular surgery facilities) [1, 9–11]. Also, the latest version of the Japanese guideline recommends (class IIa recommendation) that primary PCI for ST-segment elevation myocardial infarction (STEMI) should be performed at centers without onsite backup cardiac surgery facilities (level of evidence, B) [12, 13]. In complex elective cases, the European Society of Cardiology (ESC) guideline on myocardial revascularization mentioned that non-emergency high-risk PCI procedures should only be performed by adequately experienced operators at centers that have access to circulatory support and intensive care treatment (class IIa recommendation; level of evidence, C) [14]. Currently, PCI is an issue of concern at institutions without onsite surgical support in emergent and complex cases.

In this era, many institutions without onsite cardiac backup surgical facilities have already been established; however, it is believed that there are some differences in the experience of PCI at these institutions.

The SHINANO registry focused on the circumstances surrounding the provision of PCI at local sites in Japan, including institutions without adequately experienced operators. Therefore, it is important to further understand the outcomes of PCI at hospitals without on-site cardiac surgery facilities. The present study aimed to compare the initial and 5-year outcomes of PCI, particularly in emergent and complex elective cases, between hospitals with and without onsite cardiac surgery facilities in a single local prefecture in a mountainous area.

## Materials and methods

### Study population

**Fig. 1:**
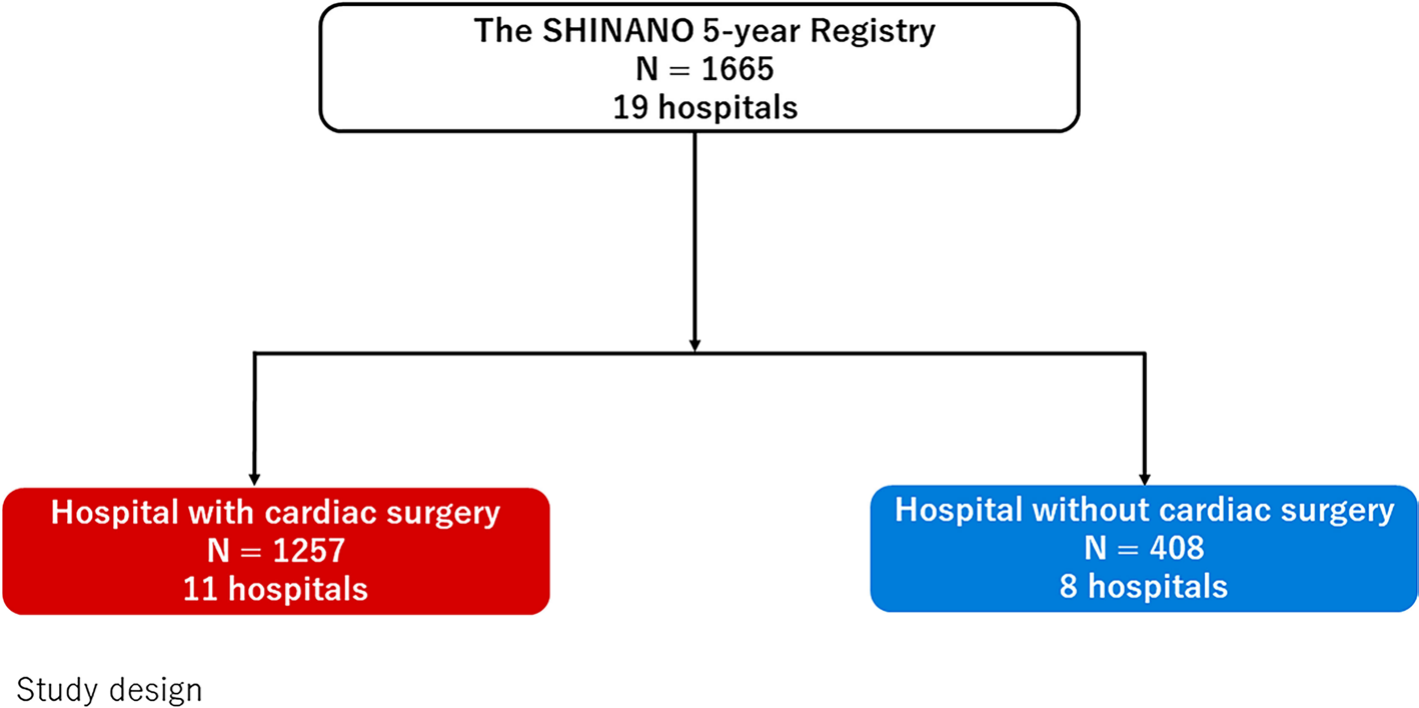
Study design

The present retrospective cohort study was based on data from the SHINANO 5-year registry from August 2012 to July 2013 obtained from the Shinshu Prospective Multicenter Analysis for Elderly Patients with Coronary Artery Disease Undergoing Percutaneous Coronary Intervention registry [9]. The SHINANO registry is a prospective, multicenter, observational registry of patients with any CAD diagnosis, including stable angina, STEMI, non-STEMI (NSTEMI), and unstable angina (UA), undergoing PCI at hospitals located in the Nagano prefecture, Japan. This study was based on an all-comer registry, and there were no exclusion criteria. The study protocol was registered with the University Hospital Medical Information Network Clinical Trials Registry, which has been approved by the International Committee of Medical Journal Editors (UMIN-ID 000010070). The study protocol was developed in accordance with the Declaration of Helsinki and was approved by the ethics committee of each participating hospital. All patients provided written informed consent before participating in this study. Among 19 collaborating hospitals in the SHINANO 5-year registry, 11 had onsite cardiac surgery facilities and 8 had no onsite cardiac surgery facilities. Of 1665 patients included in the final analysis, 408 underwent PCI at hospitals without onsite cardiac surgery facilities [onsite surgical backup (−) group] and 1257 underwent PCI at hospitals with onsite cardiac surgery facilities [onsite surgical backup (+) group]. The primary endpoint was all-cause mortality, and the secondary endpoints were major adverse cardiac and cerebrovascular events (MACCE: all-cause death, Q-wave myocardial infarction, non-fatal stroke, and target lesion revascularization) at 5 years. Of 238 patients included in the analysis regarding emergent PCI, 82 underwent PCI at hospitals without onsite cardiac surgery facilities and 156 underwent PCI at hospitals with onsite cardiac surgery facilities. Of 72 patients included in the analysis regarding complex and elective PCI, 14 underwent PCI at hospitals without onsite cardiac surgery facilities and 58 underwent PCI at hospitals with onsite cardiac surgery facilities (Fig. 1).

### Definitions

The definitions of variables were based on those of the original paper on the data emanating from the SHINANO registry [15]. Acute coronary syndrome (ACS) was a composite of STEMI, NSTEMI, and UA. “ACS positive” implied that PCI was initially performed for patients with ACS during the enrollment period. STEMI was diagnosed in patients with chest symptoms, ST-segment elevation of 1 mV in two or more limb leads, or 2 contiguous precordial leads, or left bundle branch block, and elevated biochemical markers of myocardial necrosis (troponin T level of 0.01 ng/mL or a creatine phosphokinase level twofold above the upper limit of the normal range). NSTEMI was diagnosed in patients with chest symptoms, ST-segment depression of 0.05 mV, T-wave inversion ≥ 0.3 mV, or transient ST-segment elevation < 0.05 mV, and elevated biochemical markers of myocardial necrosis (and no electrocardiogram abnormalities suggestive of STEMI). UA was diagnosed in patients with persistent resting or nocturnal chest pain with additional features. Diabetes was defined among patients with glycated hemoglobin levels of ≥ 6.5%, fasting plasma glucose levels of ≥ 126 mg/dL, or undergoing treatment with hypoglycemic agents. Hypertension was defined as a systolic blood pressure (BP) ≥ 140 mmHg, diastolic BP ≥ 90 mmHg, or receipt of therapy for hypertension. Dyslipidemia was defined as a serum total cholesterol concentration of ≥ 220 mg/dL, low-density lipoprotein cholesterol concentration ≥ 140 mg/dL, or a current receipt of treatment with lipid-lowering agents. The body mass index was calculated by dividing the weight (in kg) by the square of the patient’s height (in m). Angiographic success was defined as the achievement of a minimum reduction in the stenosis diameter to less than 20% with grade 3 Thrombolysis in Myocardial Infarction flow. The onsite surgical backup (+) group included patients treated at hospitals with full-time physicians who could perform cardiac surgery. Emergent PCI was defined as urgent angioplasty with stenting to open an infarct-related artery during an acute myocardial infarction with ST-segment elevation. Complex elective PCI was defined as a PCI procedure indicated for left main trunk disease, a single remaining patent coronary artery, and chronic total occlusions [14].

### Statistical analysis

The normality of distributions was assessed using the Shapiro–Wilk test. Continuous data were reported as the mean ± standard deviation and compared using *t* tests. Continuous variables without normal distributions were expressed as the median (interquartile range) and compared using the Mann–Whitney *U* test. Categorical variables were reported as frequencies and percentages. The characteristics of patients in the two groups were compared using chi-squared tests categorical variables or the Kruskal–Wallis test for continuous variables. Survival analyses and MACCE analyses were performed using the Kaplan–Meier method. The statistical analyses were performed using SPSS Statistics version 25.0 (IBM Corporation, Chicago, IL, USA). Analysis items with *p* < 0.05 were considered statistically significant.

## Results

### Baseline characteristics

The baseline characteristics and risk factors of the study participants are shown in Table 1. There were no differences in mean age between the two groups (70.61 ± 11.0 vs. 70.86 ± 10.7 years, *p* = 0.69) and female sex prevalence (22.8% vs. 25.7%, *p* = 0.23). There were no significant differences in the distributions of coronary risk factors and comorbidities (hypertension, dyslipidemia, and diabetes mellitus) between the groups. In addition, the groups were similar in terms of the distributions of history of heart failure, prior stroke, and previous myocardial infarction. Hemodialysis, peripheral vascular disease, and history of coronary artery bypass grafting (CABG) were more frequently observed in the onsite surgical backup (+) group than in the onsite surgical backup (−) group (7.63% vs. 2.45%, *p* < 0.01; 12.1% vs. 6.9%, *p* < 0.01; and 9.5% vs. 3.4%, *p* < 0.01, respectively). On the other hand, the rates of emergency PCI and STEMI were higher in hospitals without onsite surgical backup (40.8% vs. 51.7%, *p* < 0.01; 24.9% vs. 39.2%, *p* < 0.01, respectively).

**Table 1:** Comparison of background characteristics between onsite surgical backup (+) and onsite surgical backup (−) groups

Characteristics	Overall *N* = 1665	Surgical cover (+) *N* = 1257	Surgical cover (−) *N* = 408	*P* value
Age, years	70.7 ± 10.9	70.6 ± 11.0	70.8 ± 10.7	0.69
Age ≥ 75 years	667 (40.0)	505 (40.2)	162 (39.7)	0.91
Women	392 (23.5)	287 (22.8)	105 (25.7)	0.23
Body mass index (kg/m^2^)	23.3 ± 7.36	23.2 ± 8.13	23.56 ± 3.89	0.40
Hypertension	1237 (74.3)	934 (74.3)	303 (74.3)	1.00
Dyslipidemia	1008 (60.5)	758 (60.4)	250 (61.3)	0.77
Smoking (current or former)	1033 (62.0)	781 (62.1)	252 (61.3)	0.91
Diabetic mellitus	608 (36.5)	474 (37.7)	134 (32.8)	0.14
Hemodialysis	106 (6.4)	96 (7.6)	10 (2.45)	< 0.001
History of heart failure	208 (12.5)	165 (13.1)	43 (10.5)	0.28
Ejection fraction, %	59.9 ± 13.6	60.5 ± 13.8	58.4 ± 12.8	0.01
History of stroke	176 (10.6)	137 (10.9)	39 (9.56)	0.52
Peripheral vascular disease	180 (10.8)	152 (12.1)	28 (6.9)	< 0.001
Previous myocardial infarction	419 (25.2)	328 (26.1)	91 (22.3)	0.07
Previous PCI	396 (23.8)	297 (24.2)	99 (24.8)	0.09
Previous CABG	133 (8.0)	119 (9.5)	14 (3.4)	< 0.001
*Clinical status at the time of PCI*				
Elective PCI	941 (56.5)	744 (59.2)	197 (48.3)	< 0.001
Primary PCI	724 (43.5)	513 (40.8)	211 (51.7)	< 0.001
STEMI	473 (28.4)	313 (24.9)	160 (39.2)	< 0.001
NSTEMI	93 (5.6)	70 (5.57)	23 (5.64)	0.9
Unstable angina	165 (9.9)	108 (8.6)	57 (13.0)	< 0.01

Data are presented as median and interquartile range or *n* (%)

PCI, percutaneous coronary intervention; CABG, coronary artery bypass grafting; STEMI, ST elevation myocardial infarction; NSTEMI, non-ST-segment elevation myocardial infarction

There were no significant differences in the distribution of patient backgrounds and risk factors between the emergent PCI group and the complex elective PCI group with and without surgery.

### Lesion characteristics, procedures, and complications

Table 2 shows the distributions of lesion characteristics, procedures, and PCI complications. In terms of lesion difficulty, number of diseased vessels, bifurcation lesions, chronic total occlusions, and Synergy Between Percutaneous Coronary Intervention with Taxus and Cardiac Surgery scores, no significant differences were found between the two groups. Regarding the technical aspects of PCI, the rates of use of drug-eluting stents and multiple stents were significantly higher in the onsite surgical backup (+) group (46.9% vs. 37.5%, *p* < 0.01; 22.2% vs. 13.5%, *p* < 0.01, respectively). However, the rates of use of intra-aortic balloon pumping during PCI were significantly higher in the onsite surgical backup (−) group (4.77% vs. 7.84%, *p* = 0.024). The rate of complete revascularization was higher in the onsite surgical backup (+) group than in the surgical backup (−) group (67.5% vs. 59.3%, *p* < 0.01).

**Table 2:** Comparison of lesion characteristics between onsite surgical backup (+) and surgical backup (−) groups

Lesion characteristics	Surgical cover (+) *N* = 1257	Surgical cover (−) *N* = 408	*P* value
*Approach*			
TRI	799 (63.6)	242 (59.3)	0.13
TBI	43 (3.42)	18 (4.41)	0.36
TFI	409 (32.5)	114 (27.9)	0.09
*Distribution*			
1 vessel	791 (62.9)	242 (59.3)	0.13
2 vessels	307 (24.4)	117 (28.7)	0.36
3 vessels	158 (12.6)	49 (12.0)	0.09
*Location of vessel*			
Left main coronary artery	28 (2.23)	6 (1.47)	0.61
Left anterior descending artery	565 (44.9)	179 (43.9)	0.66
Circumflex artery	202 (16.1)	64 (4.0)	0.37
Right coronary artery	444 (35.3)	157 (38.5)	0.43
Graft artery	6 (4.77)	0 (0)	0.25
TIMI grade 3	1167 (92.8)	367 (90.0)	0.06
Chronic total occlusion	82 (6.5)	18 (4.4)	0.15
Bifurcation	340 (27.0)	127 (31.1)	0.11
SYNTAX score	12.8 ± 9.1)	13.0 ± 8.6	0.73
Multiple stent	279 (22.2)	55 (13.5)	< 0.001
*Type of stent*			
Bare-metal stent	455 (36.2)	177 (43.4)	0.01
Drug-eluting stent	589 (46.9)	153 (37.5)	< 0.001
Requirement of IABP	60 (4.77)	32 (7.84)	0.02
Requirement of ECMO	12 (0.95)	6 (1.47)	0.41
Only POBA	150 (11.9)	64 (15.7)	0.12
*Complications*			
Coronary dissection	78 (6.2)	18 (4.4)	0.09
Coronary perforation	16 (1.27)	1 (0.25)	0.09
Complete revascularization	848 (67.5)	242 (59.3)	0.01

Data are presented as median and interquartile range or *n* (%)

TRI, trans-radial intervention; TBI, trans-brachial intervention; TFI, trans-femoral intervention; TIMI, thrombolysis in myocardial infarction trial; IABP, intra-aortic balloon pumping; ECMO, extracorporeal membrane oxygenation; POBA, plain old balloon angioplasty

### Initial and long-term outcomes

The initial success rates were similar between the onsite surgical backup (+) and surgical backup (−) groups (92.8% vs. 90.0%, *p* = 0.08), despite differences in lesion complexity. There were no differences in in-hospital mortality rate between the two groups (2.07% vs. 3.68%, *p* = 0.095). In terms of each complication related to PCI, the rates of stent thrombosis and PCI-related stroke were similar between the two groups (0.32% vs. 0.00%, *p* = 0.578; 0.32% vs. 0.74%, *p* = 0.372, respectively). In contrast, the rate of in-hospital bleeding events was significantly higher in the onsite surgical backup (−) group (0.00% vs. 2.70%, *p* < 0.001). There was no difference in the number of cases requiring emergent CABG between the two groups (0.24% vs. 0.25%, *p* = 1.00) (Table 2).

**Fig. 2:**
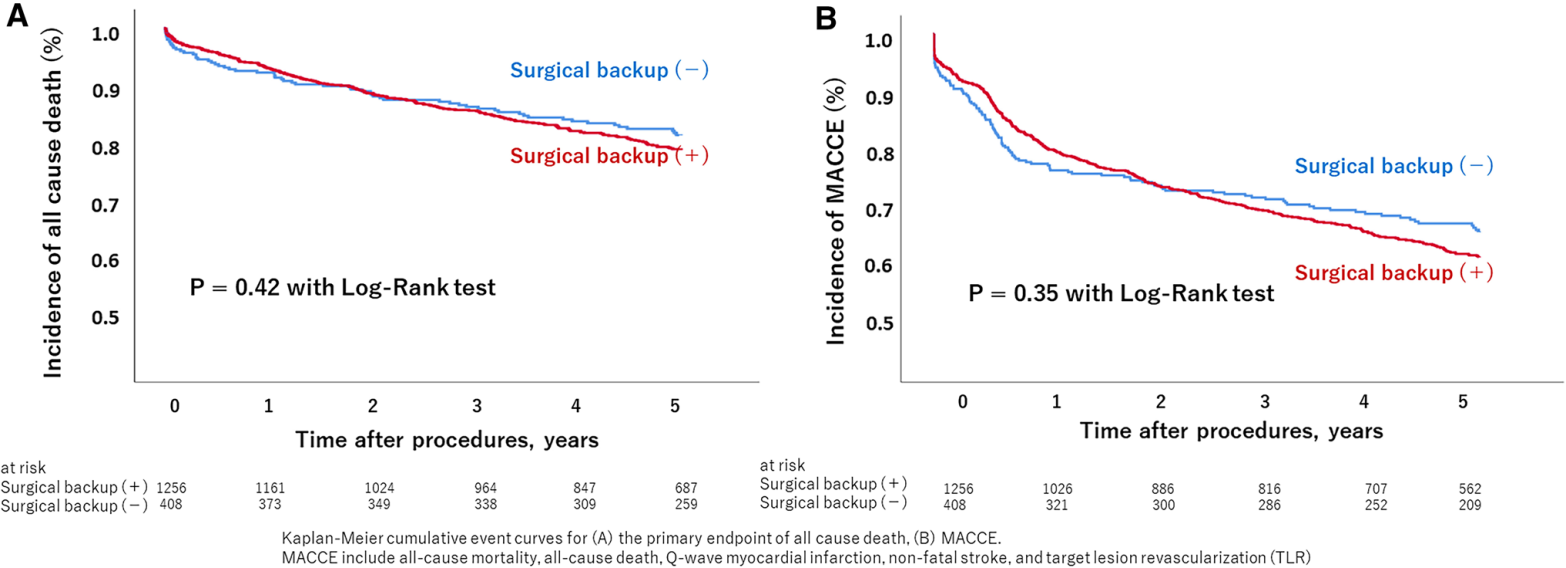
Kaplan–Meier cumulative event curves for **A** the primary endpoint of all cause death, **B** MACCE, MACCE include all-cause death, Q-wave myocardial infarction, non-fatal stroke, and target lesion revascularization (TLR)

In the 5-year analysis, there were no differences in the rates of all-cause mortality, Q-wave myocardial infarction, and non-fatal stroke (16.4% vs. 15.2%, *p* = 0.35; 4.22% vs. 15.2%, *p* = 0.23; and 4.46% vs. 4.90%, *p* = 0.82, respectively) (Table 3). The Kaplan–Meier analysis showed no differences between the two groups in terms of overall survival (31.6% vs. 28.9%, *p* = 0.354) and MACCE (22.2% vs. 20.1%, *p* = 0.269) (Fig. 2).

**Table 3:** Comparison of initial and long-term outcomes between onsite surgical backup (+) and surgical backup (−) groups

Adverse events	Surgical cover (+) *N* = 1257	Surgical cover (−) *N* = 408	*P* value
*Initial outcomes*			
Mortality	26 (2.07)	15 (3.68)	0.095
Stent thrombosis	4 (0.32)	0 (0.00)	0.578
Stroke	4 (0.32)	3 (0.74)	0.372
Bleeding event	0 (0.00)	11 (2.70)	0.000
Requirement of emergency CABG	3 (0.24)	1 (0.25)	1
*Long-term outcomes*			
Major adverse cardiac events	397 (31.5)	118 (28.9)	0.35
All-cause death	206 (16.4)	62 (15.2)	0.42
Cardiac death	133 (10.6)	41 (10.0)	0.85
Q-wave myocardial infarction	53 (4.22)	12 (15.2)	0.23
Non-fatal stroke	56 (4.46)	20 (4.90)	0.82
Target lesion revascularization	148 (11.8)	43 (10.5)	0.57
CABG	16 (1.27)	8 (1.96)	0.34
Bleeding event	80 (6.36)	35 (8.58)	0.14

Data are presented as *n* (%)

CABG, coronary artery bypass grafting

### Long-term outcomes of emergent and complex elective cases

**Fig. 3:**
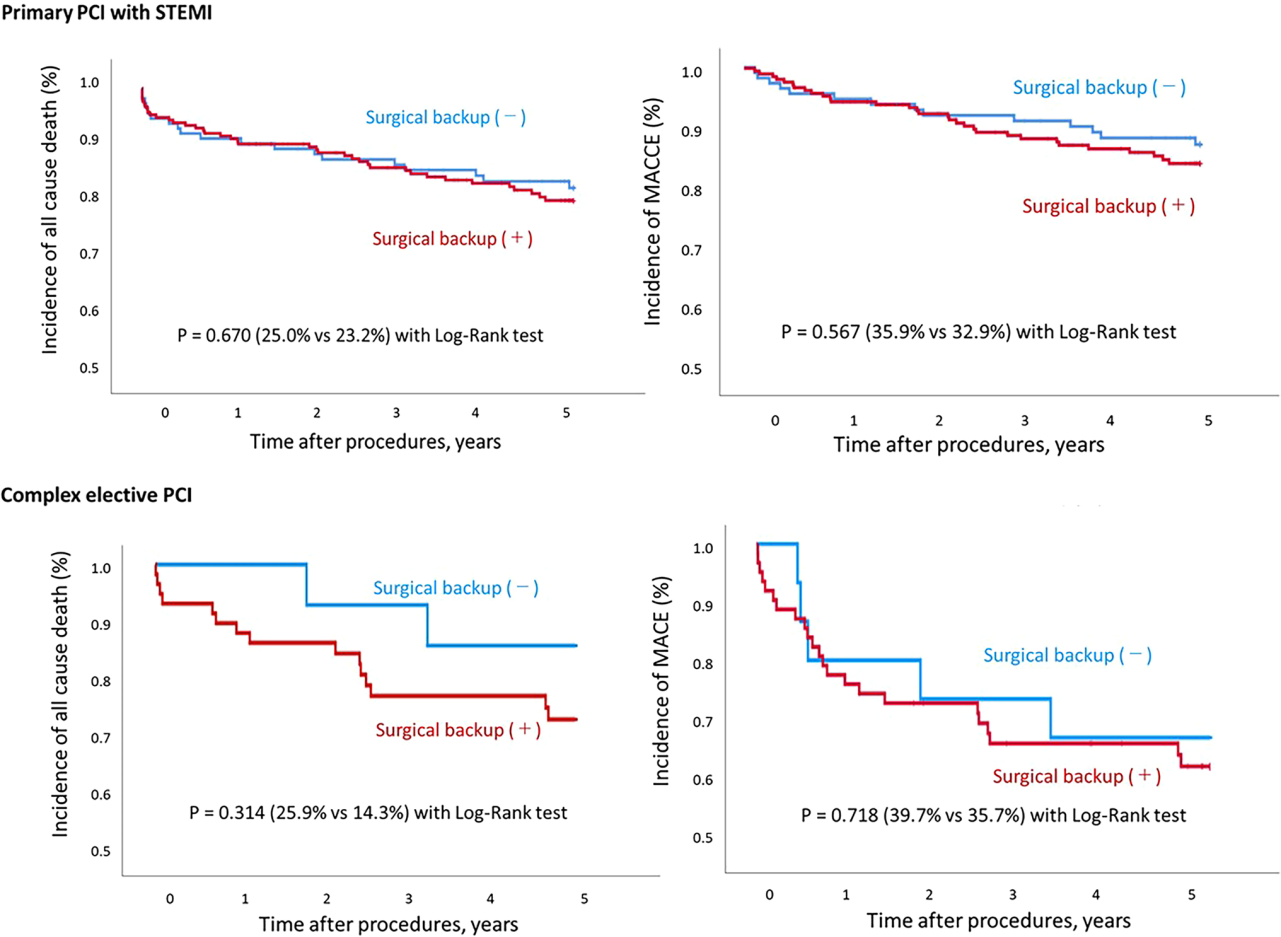
Kaplan–Meier cumulative event curves for all cause death and MACCE in primary PCI and complex elective PCI. MACCE include all-cause death, Q-wave myocardial infarction, non-fatal stroke and target lesion revascularization (TLR)

The Kaplan–Meier analysis showed no differences between the two groups in terms of overall survival (25.0% vs. 23.2%, *p* = 0.670) and MACCE (35.9% vs. 32.9%, *p* = 0.567) (Fig. 3) among emergent PCI cases. Also, among complex elective cases, there were no differences between the two groups in terms of overall survival (25.9% vs. 14.3%, *p* = 0.314) and MACCE (39.7% vs. 35.7%, *p* = 0.718) (Fig. 3).

## Discussion

We have shown that there were no differences in initial outcome, long-term mortality, and the incidences of MACCE between patients who underwent PCI at hospitals with onsite surgical backup facilities and those who underwent PCI at hospitals without onsite surgical backup facilities. Also, among patients who underwent emergent PCI on account of STEMI and complex elective PCI there were no significant differences between two groups.

Over a decade ago, in-hospital and 30-day mortality rates were higher in centers without onsite cardiac surgery facilities compared to those with onsite surgical backup facilities [16]. Indeed, the guidelines of those days did not recommend both elective and emergent PCI in institutions without onsite cardiac surgical backup facilities [17]. That recommendation was mainly made to prevent acute occlusions; however, these problems have been resolved in the present decade [2].

Indeed, PCI in hospitals without onsite surgical backup facilities have yielded some favorable results, and the complications of PCI and the need for emergency CABG have become rare events, occurring at rates of only 0.3% [18] to 0.7% [19]. Emergent PCI for STEMI was shown to yield similar outcomes in hospitals with or without cardiac surgery facilities in the presence of an experienced operator [20].

In the last few years, large meta-analyses have reported no significant differences in mortality rates between the two types of institutions (in terms of the presence of cardiovascular surgery for emergent PCI) [1, 9]. In response to these trends, the Japanese guideline recommended that emergent PCI for STEMI should be performed at centers without onsite backup cardiac surgery facilities (class IIa recommendation; level of evidence, B).

A previous study compared the delay of revascularization between emergent PCI performed at an institution without onsite cardiac surgery facilities and emergent PCI performed after transfer to a hospital with onsite cardiac surgery facilities, and revealed a delay of approximately 60 min between the two groups [21]. In general, myocardial infarction is an emergent condition [22] and it is recommended that emergent PCI should be performed as quickly as possible for STEMI within 12 h of onset [23, 24]. In this era, a reduction in the delay of transfer for many institutions that provide PCI might be required.

On the other hand, in complex elective cases, the ESC guideline on myocardial revascularization mentioned that non-emergency high-risk PCI procedures should only be performed by adequately experienced operators at centers that have the facilities to provide circulatory support and intensive care treatment (class IIa recommendation; level of evidence, C) [14]. However, sometimes it is difficult to transfer patients between facilities due to the patient’s condition and the location of facilities. Although the number of complex elective cases were low in this study population, our data supported the safety and efficacy of PCI for emergent cases or complex elective cases.

Considering recent improvements in the quality of PCI, the establishment of a consulting system with experienced operators might enable PCI to be performed for complex cases at institutions without onsite backup surgical facilities. In that respect, establishing a local system and consultation protocol for each situation (e.g., according to emergency and complexity) are important to maintain the safety of patients with CAD.

The main finding of the present study was that the onsite surgical backup (−) group was non-inferior in terms of both acute and long-term outcomes. Furthermore, this non-inferiority was also observed among emergent and/or complex cases. This result might reflect the procedural establishment of PCI, as mentioned above. In addition, it suggests that institutions that provide PCI can function without onsite surgical support in this era. These findings are novel and important for the assessment of the current local medical situation. We also believe that it is useful to assess an appropriate comprehensive system of treatment for patients with CAD. However, further studies should be required to examine the prognosis of the patients who underwent emergent PCI or complex elective PCI, because our research was in one local prefecture and study population was small.

Several limitations of this study should be acknowledged. First, this study was a retrospective analysis. Second, the selection of strategy (between PCI and CABG) entirely depended on each physician’s discretion, which might have led to some bias in our analysis. Third, more than 40% of patients underwent bare-metal stent placement, based on which the strategy may not apply to patients with CAD in clinical practice. Fourth, the cooperation and consultation between each institution were ambiguous. Finally, the experience of PCI operators in each institution was different, which might have caused some discrepancy in the selection of strategy and the outcomes of the procedures.

Emergency PCI is performed more frequently at institutions without onsite backup surgical facilities. There are no differences in the acute and long-term outcomes of PCI between institutions with and without onsite backup surgical facilities. Particularly, regarding PCI for both emergent and complex elective cases, there were no differences in initial and long-term outcomes between the two groups. In this era, even institutions without onsite cardiac surgical backup facilities may be able to provide appropriate care to patients with CAD.
